# Supplementary material for: Characterization of the Complete Mitochondrial Genome of Agelas nakamurai from the South China Sea
Source: Int J Mol Sci. 2023 Dec 26;25(1):357. doi: 10.3390/ijms25010357 (PMC10779334; doi:10.3390/ijms25010357)
Supplement: Supplementary file 1 [file ijms-25-00357-s001.zip › ijms-2723548-supplementary.pdf]

Supplementary Tables and Figures

Supplementary Table S1. Detailed annotation information of *Agelas nakamurai* mitochondrial genome.

Feature	Nucleotide size (bp)	Position	Product	Start codon	Stop codon
cox1	1569	1-1569	cytochrome c oxidase subunit 1	ATG	TAA
tRNA-Ser (TGA)	80	1647-1726	tRNA-Ser		
tRNA-Ala (TGC)	72	1740-1811	tRNA-Ala		
tRNA-Cys (GCA)	72	1850-1921	tRNA-Cys		
nad1	975	2022-2996	NADH dehydrogenase subunit 1	ATG	TAG
tRNA-Leu (TAA)	71	3083-3153	tRNA-Leu		
tRNA-Leu (TAG)	72	3265-3336	tRNA-Leu		
tRNA-Tyr (GTA)	72	3359-3430	tRNA-Tyr		
tRNA-Met (CAT)	72	3439-3510	tRNA-Met		
tRNA-Met (CAT)	71	3537-3607	tRNA-Met		
nad2	1461	3669-5129	NADH dehydrogenase subunit 2	ATG	TAA
nad5	1866	5170-7035	NADH dehydrogenase subunit 5	ATG	TAA
tRNA-Ile (GAT)	71	7077-7147	tRNA-Ile		
tRNA-Phe (GAA)	71	7186-7256	tRNA-Phe		
rns	1275	7257-8531	s-rRNA		
tRNA-Gly (TCC)	72	8532-8603	tRNA-Gly		
tRNA-Val (TAC)	73	8605-8677	tRNA-Val		
rnl	2587	8784-11370	l-rRNA		
cox2	744	11782-12525	cytochrome c oxidase subunit 2	ATG	TAA
atp8	285	12575-12859	ATP synthase F0 subunit 8	ATG	TAA
atp6	735	12987-13721	ATP synthase F0 subunit 6	GTG	TAA
tRNA-Arg (TCT)	73	13845-13917	tRNA-Arg		
cox3	786	13994-14779	cytochrome c oxidase subunit 3	ATG	TAA
tRNA-Gln (TTG)	73	14838-14910	tRNA-Gln		
tRNA-Trp (TCA)	71	14950-15020	tRNA-Trp		
cob	1146	15092-16237	cytochrome b	ATG	TAG
tRNA-Thr (TGT)	72	16330-16401	tRNA-Thr		
atp9	237	16549-16785	ATP synthase F0 subunit 9	ATG	TAA
tRNA-Ser (GCT)	84	16990-17073	tRNA-Ser		
tRNA-Lys (TTT)	73	17101-17173	tRNA-Lys		
tRNA-Asn (GTT)	71	17239-17309	tRNA-Asn		
tRNA-Met (CAT)	71	17377-17447	tRNA-Met		
tRNA-His (GTG)	73	17456-17528	tRNA-His		
tRNA-Pro (TGG)	72	17569-17640	tRNA-Pro		
nad4	1470	17661-19130	NADH dehydrogenase subunit 4	ATG	TAA
tRNA-Glu (TTC)	73	19184-19256	tRNA-Glu		

nad6	573	19254-19826	NADH dehydrogenase subunit 6	GTG	TAA
nad3	357	19919-20275	NADH dehydrogenase subunit 3	ATG	TAG
tRNA-Arg (TCG)	71	20474-20544	tRNA-Arg		
nad4L	300	20545-20844	NADH dehydrogenase subunit 4L	ATG	TAA

Supplementary Table S2. Detailed information of 38 Demospongiae sponge mitochondrial genomes for phylogenetic analysis.

Accession	Organism	Order	Superfamily	Family	Genus	Length	AT%
OQ363829	*Agelas nakamurai*	Agelasida	Agelasida	Agelasidae	Agelas	20885	62.4
NC_010213.1	*Agelas schmidtii*	Agelasida	Agelasida	Agelasidae	Agelas	20360	61.7
NC_008944.1	*Amphimedon queenslandica*	Haplosclerida	Haplosclerida	Niphatidae	Amphimedon	19960	56.1
NC_010203.1	*Aplysina fulva*	Verongiida	Verongiida	Aplysinidae	Aplysina	19620	67.1
NC_053937.1	*Aplysina gerardogreeni*	Verongiida	Verongiida	Aplysinidae	Aplysina	19620	67.2
NC_006894.1	*Axinella corrugata*	Axinellida	Axinellida	Axinellidae	Axinella	25610	57.1
JQ302310.1	*Baikalospongia intermedia*	Spongillida	Spongillida	Lubomirskiidae	Baikalospongia	28227	58.8
NC_031192.1	*Cacospongia mycofijiensis*	Dictyoceratida	Dictyoceratida	Thorectidae	Cacospongia	16227	62.9
NC_010206.1	*Callyspongia plicifera*	Haplosclerida	Haplosclerida	Callyspongiidae	Callyspongia	18846	64.3
NC_010208.1	*Chondrilla aff. nucula CHOND*	Chondrillida	Chondrillida	Chondrillidae	Chondrilla	19282	66.4
NC_010198.1	*Cinachyrella kuekenthali*	Tetractinellida	Tetractinellida	Tetillidae	Cinachyrella	18089	64
NC_069646.1	*Cliona patera*	Clionaida	Clionaida	Clionaidae	Cliona	19133	67
NC_027520.1	*Crella elegans*	Poecilosclerida	Poecilosclerida	Crellidae	Crella	18543	63.9
NC_010210.1	*Ectyoplasia ferox*	Axinellida	Axinellida	Raspailiidae	Ectyoplasia	18312	69.5
NC_010202.1	*Ephydatia muelleri*	Spongillida	Spongillida	Spongillidae	Ephydatia	23929	59.6
NC_016431.1	*Eunapius subterraneus*	Spongillida	Spongillida	Spongillidae	Eunapius	24850	59.2
NC_006990.1	*Geodia neptuni*	Tetractinellida	Tetractinellida	Geodiidae	Geodia	18020	66.5
NC_037391.1	*Halichondria okadai*	Suberitida	Suberitida	Halichondriidae	Halichondria	20722	64.2
NC_014876.1	*Halisarca harmelini*	Dendroceratida	Dendroceratida	Halisarcidae	Halisarca	20591	65.4
EU237484.1	*Hippospongia lachne*	Dictyoceratida	Dictyoceratida	Spongiidae	Hippospongia	16755	63
NC_021422.1	*Hyattella sinuosa*	Dictyoceratida	Dictyoceratida	Spongiidae	Hyattella	16422	62.7
NC_022450.1	*Hymeniacidon sinapium*	Suberitida	Suberitida	Halichondriidae	Hymeniacidon	23435	65.5
NC_010216.1	*Igernella notabilis*	Dendroceratida	Dendroceratida	Dictyodendrillidae	Igernella	20310	72
NC_010207.1	*Iotrochota birotulata*	Poecilosclerida	Poecilosclerida	Iotrochotidae	Iotrochota	19112	64.4
NC_013662.1	*Ircinia strobilina*	Dictyoceratida	Dictyoceratida	Irciniidae	Ircinia	16414	63
NC_013760.1	*Lubomirskia baikalensis*	Spongillida	Spongillida	Lubomirskiidae	Lubomirskia	28958	58.5
KR911863.1	*Petrosia ficiformis*	Haplosclerida	Haplosclerida	Petrosiidae	Petrosia	18887	63.7

NC_025335.1	*Poecillastra laminaris*	Tetractinellida	Tetractinellida	Vulcanellidae	Poecillastra	18413	64.3
NC_023834.1	*Polymastia littoralis*	Polymastiida	Polymastiida	Polymastiidae	Polymastia	21719	69
NC_010209.1	*Ptilocaulis walpersi*	Axinellida	Axinellida	Axinellidae	Ptilocaulis	18865	71.4
NC_018360.1	*Rezinkovia echinata*	Spongillida	Spongillida	Lubomirskiidae	Rezinkovia	28614	58.7
NC_010496.1	*Suberites domuncula*	Suberitida	Suberitida	Suberitidae	Suberites	26300	64.5
NC_065020.1	*Terpios hoshinota*	Suberitida	Suberitida	NA	Terpios	20504	63.5
NC_006991.1	*Tethya actinia*	Tethyida	Tethyida	Tethyidae	Tethya	19565	70.6
MN587873.1	*Thoosa mismalolli*	Tetractinellida	Tetractinellida	Thoosidae	Thoosa	19019	66.7
NC_010204.1	*Topsentia ophiraphidites*	Suberitida	Suberitida	NA	Topsentia	19763	66.3
NC_010218.1	*Vaceletia* sp.	Dictyoceratida	Dictyoceratida	Verticillitidae	Vaceletia	20658	70.6
NC_010211.1	*Xestospongia muta*	Haplosclerida	Haplosclerida	Petrosiidae	Xestospongia	18990	66.1

Supplementary Table S3. Information on 17 mitochondrial genomes of Demospongiae sponges for evolutionary analysis.

Accession	Organism	Order	Superfamily	Family	Genus	Length	AT%
OQ363829	*Agelas nakamurai*	Agelasida	Agelasida	Agelasidae	Agelas	20885	62.4
EU237475.1	*Agelas schmidtii*	Agelasida	Agelasida	Agelasidae	Agelas	20360	61.7
EU237474.1	*Amphimedon compressa*	Haplosclerida	Haplosclerida	Niphatidae	Amphimedon	18564	59.2
EU237477.1	*Callyspongia plicifera*	Haplosclerida	Haplosclerida	Callyspongiidae	Callyspongia	18846	64.3
EU237479.1	*Cinachyrella kuekenthali*	Tetractinellida	Tetractinellida	Tetillidae	Cinachyrella	18089	64
KR911862.1	*Crella elegans*	Poecilosclerida	Poecilosclerida	Crellidae	Crella	18543	63.9
EU237480.1	*Ectyoplasia ferox*	Axinellida	Axinellida	Raspailiidae	Ectyoplasia	18312	69.5
EU237481.1	*Ephydatia muelleri*	Spongillida	Spongillida	Spongillidae	Ephydatia	23929	59.6
AY320032.1	*Geodia neptuni*	Tetractinellida	Tetractinellida	Geodiidae	Geodia	18020	66.5
MH756603.1	*Halichondria panicea*	Suberitida	Suberitida	Halichondriidae	Halichondria	19571	63.2
EU237486.1	*Iotrochota birotulata*	Poecilosclerida	Poecilosclerida	Iotrochotidae	Iotrochota	19112	64.4
KR911863.1	*Petrosia ficiformis*	Haplosclerida	Haplosclerida	Petrosiidae	Petrosia	18887	63.7
EU237488.1	*Ptilocaulis walpersi*	Axinellida	Axinellida	Axinellidae	Ptilocaulis	18865	71.4
AY320033.1	*Tethya actinia*	Tethyida	Tethyida	Tethyidae	Tethya	19565	70.6
MN587873.1	*Thoosa mismalolli*	Tetractinellida	Tetractinellida	Thoosidae	Thoosa	19019	66.7
EU237482.1	*Topsentia ophiraphidites*	Suberitida	Suberitida	NA	Topsentia	19763	66.3
EU237490.1	*Xestospongia muta*	Haplosclerida	Haplosclerida	Petrosiidae	Xestospongia	18990	66.1

Supplementary Table S4. Best partition scheme and model for BI phylogenetic analysis.

Subset partitions	Best model
P1: (atp6, cox2, cox3, nad1)	GTR+F+I+G4
P2: (atp8)	HKY+F+I+G4
P3: (atp9)	GTR+F+I+G4
P4: (cox1)	GTR+F+I+G4
P5: (cytb, nad2)	GTR+F+I+G4
P6: (nad3, nad4L, nad4, nad5, nad6)	GTR+F+I+G4

Supplementary Table S5. Best partition scheme and model for ML phylogenetic analysis.

Subset partitions	Best model
P1: (atp6, cox3)	GTR+F+R4
P2: (atp8)	TPM3u+F+I+I+R3
P3: (atp9)	TIM+F+I+G4
P4: (cox1)	TIM2+F+I+G4
P5: (cox2, nad1, nad3, nad4, nad5, nad6)	GTR+F+I+I+R4
P6: (cytb, nad2, nad4L)	GTR+F+I+I+R4

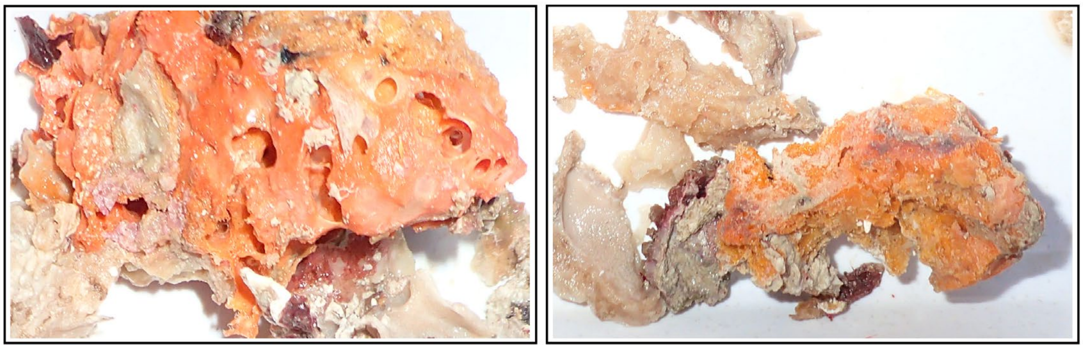

Supplementary Figure S1. Sponge tissue sample placed on the dissection tray.

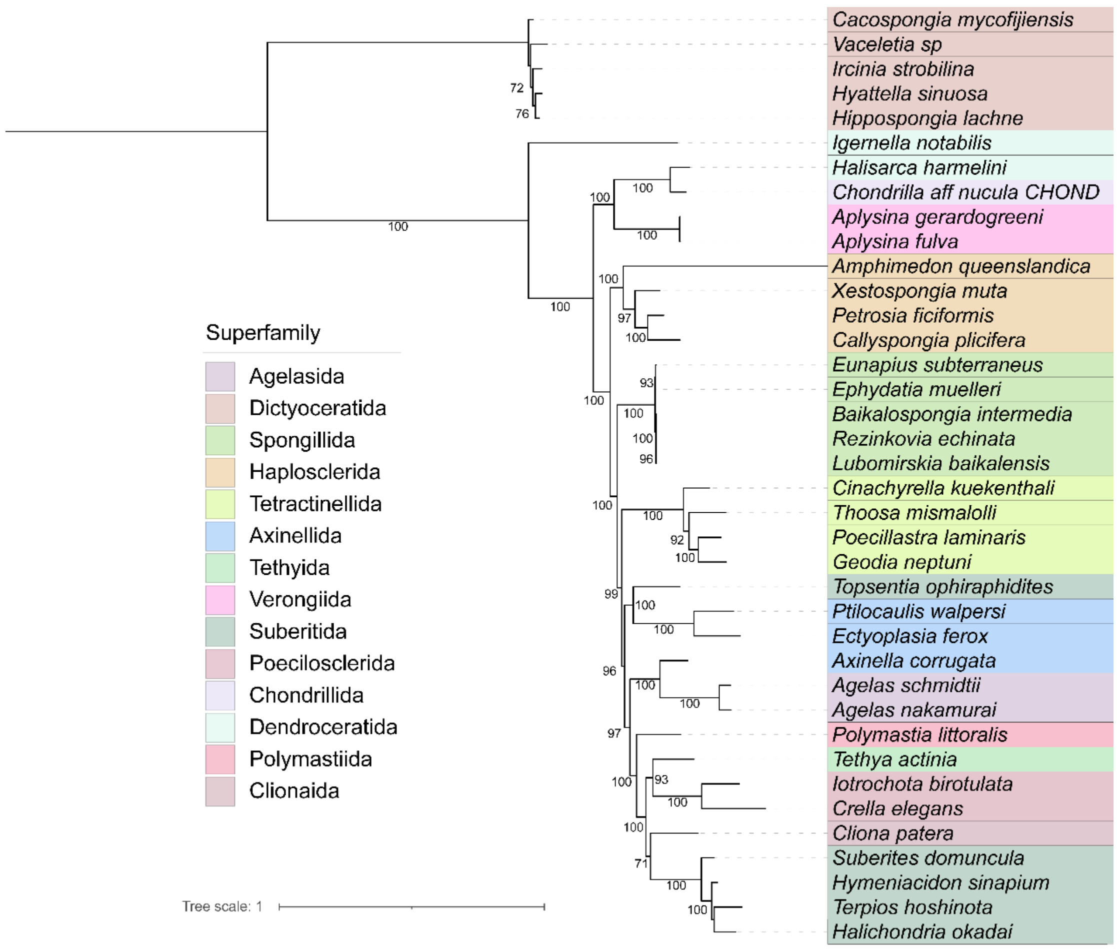

Supplementary Figure S2. ML phylogenetic tree of 38 Demospongiae sponges constructed using mitochondrial genome PCGs. *Hippospongia lachne*, *Hyattella sinuosa*, *Ircinia strobilina*, *Vaceletia* sp. and *Cacospongia mycofijiensis* were used as outgroups for tree construction. The numbers adjacent to the nodes indicate the percent posterior probability values, with only values exceeding 70 being shown. Leaf labels are color-coded to represent different superfamilies.

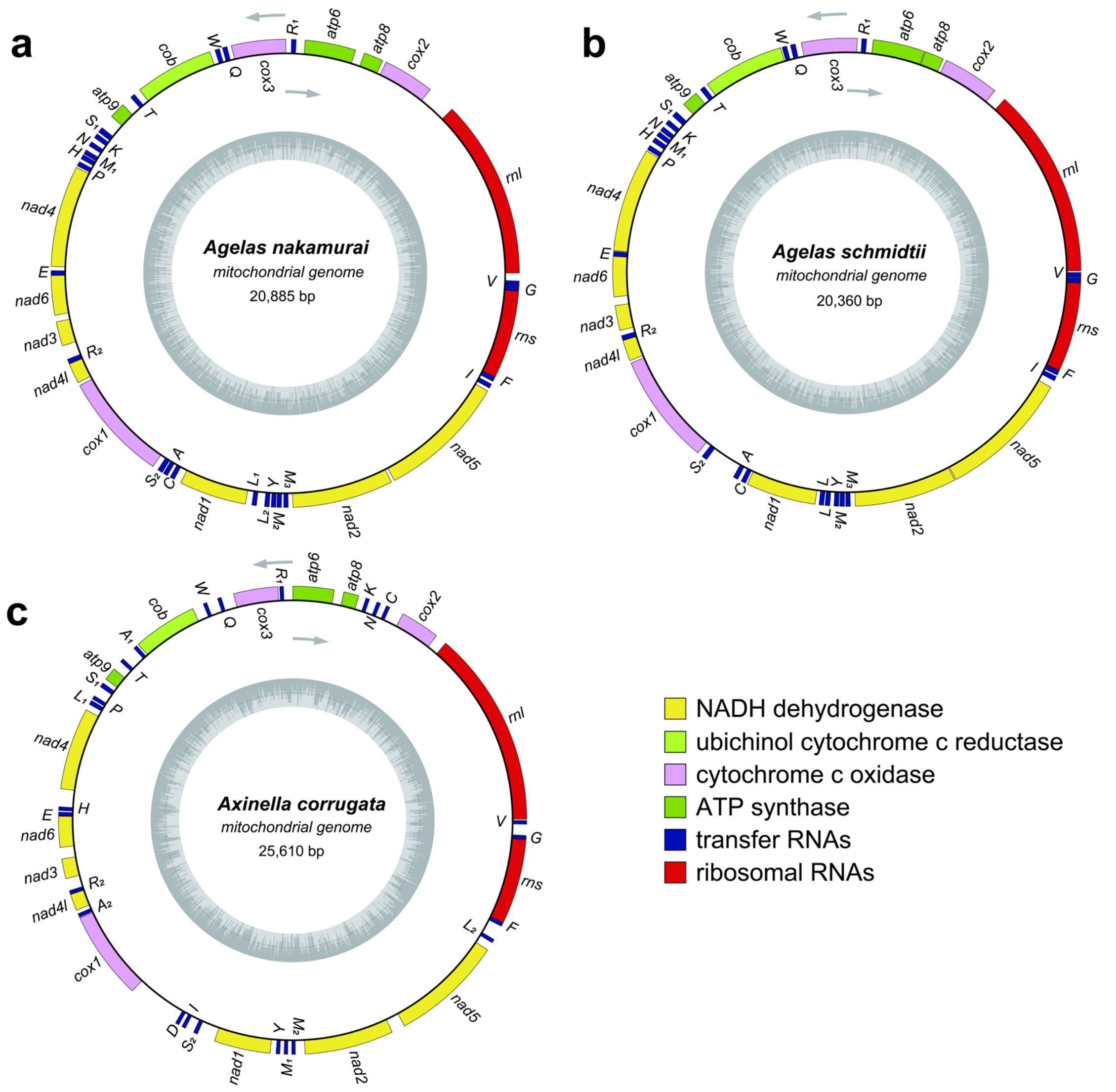

Supplementary Figure S3. Mitogenome maps of *Agelas nakamurai*, *Agelas schmidti*, and *Axinella corrugata*. (a), (b) and (c) represent the complete mitogenome maps of *Agelas nakamurai*, *Agelas schmidti*, and *Axinella corrugata*, respectively. Distinct colors correspond to various genes. The tRNA genes are denoted by single-letter abbreviations representing the accepted amino acid. The gray inner ring represents the GC content of the sequence.

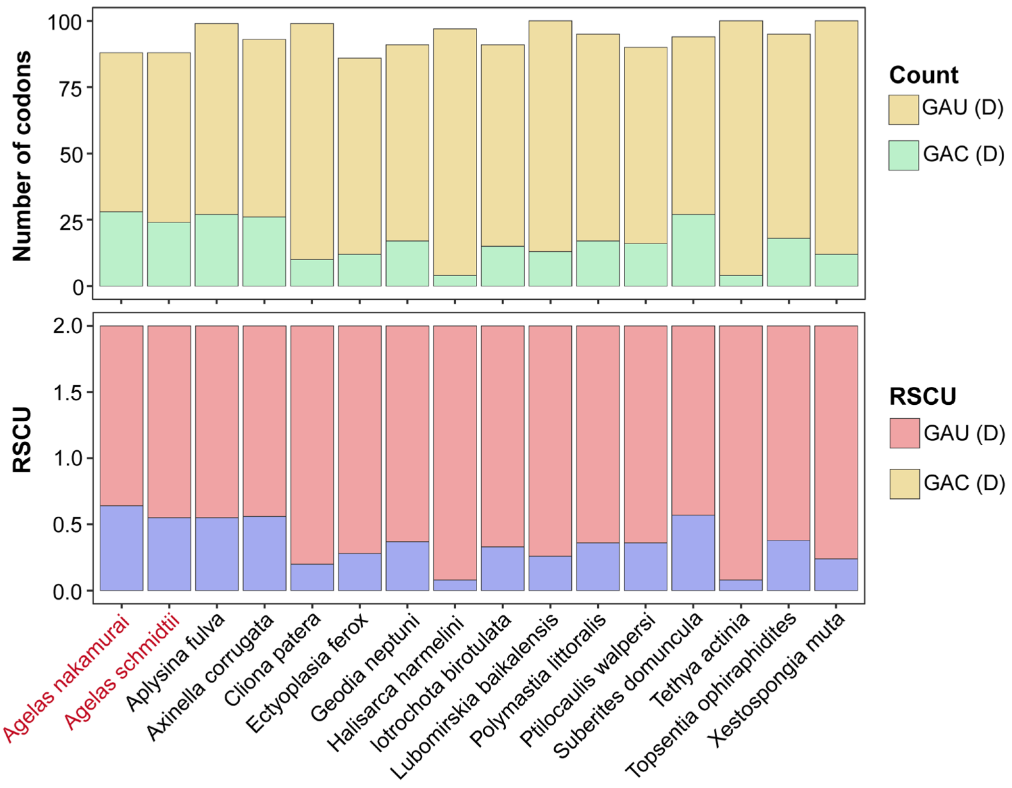

Supplementary Figure S4. Number of aspartic acid codons and RSCU in 16 Demospongiae mitochondrial PCGs.
